# Interferon regulatory factor 4 as a therapeutic target in adult T-cell leukemia lymphoma

**DOI:** 10.1186/s12977-020-00535-z

**Published:** 2020-08-28

**Authors:** Daniel A. Rauch, Sydney L. Olson, John C. Harding, Hemalatha Sundaramoorthi, Youngsoo Kim, Tianyuan Zhou, A. Robert MacLeod, Grant Challen, Lee Ratner

**Affiliations:** 1 Division of Oncology, Department of Medicine, Washington University in St. Louis, 660 S Euclid Ave, Box 8069, St Louis, MO 63110 USA; 2 Ionis Pharmaceuticals, Carlsbad, CA USA

**Keywords:** HTLV-1, ATLL, IRF4, Lenalidomide, ASO

## Abstract

**Background:**

Adult T-cell leukemia lymphoma (ATLL) is a chemotherapy-resistant malignancy with a median survival of less than one year that will afflict between one hundred thousand and one million individuals worldwide who are currently infected with human T-cell leukemia virus type 1. Recurrent somatic mutations in host genes have exposed the T-cell receptor pathway through nuclear factor κB to interferon regulatory factor 4 (IRF4) as an essential driver for this malignancy. We sought to determine if IRF4 represents a therapeutic target for ATLL and to identify downstream effectors and biomarkers of IRF4 signaling in vivo.

**Results:**

ATLL cell lines, particularly Tax viral oncoprotein-negative cell lines, that most closely resemble ATLL in humans, were sensitive to dose- and time-dependent inhibition by a next-generation class of IRF4 antisense oligonucleotides (ASOs) that employ constrained ethyl residues that mediate RNase H-dependent RNA degradation. ATLL cell lines were also sensitive to lenalidomide, which repressed IRF4 expression. Both ASOs and lenalidomide inhibited ATLL proliferation in vitro and in vivo*.* To identify biomarkers of IRF4-mediated CD4 + T-cell expansion in vivo*,* transcriptomic analysis identified several genes that encode key regulators of ATLL, including interleukin 2 receptor subunits α and β, KIT ligand, cytotoxic T-lymphocyte-associated protein 4, and thymocyte selection-associated high mobility group protein TOX 2.

**Conclusions:**

These data support the pursuit of IRF4 as a therapeutic target in ATLL with the use of either ASOs or lenalidomide.

## Background

Adult T-cell leukemia lymphoma (ATLL) is an incurable, chemotherapy-resistant human malignancy that will afflict approximately 5% of the 20 million people infected with human T-cell leukemia virus type 1 (HTLV-1) [1]. In the U.S, ATLL disproportionately affects minorities of Native American, Caribbean, African, and Asian heritage [2]. ATLL is derived from CD25 + CADM1 + T regulatory (Treg) cells and is characterized by blood and bone marrow involvement, hypercalcemia, and lytic bone lesions [3]. Clinical classifications of ATLL include four subtypes: a smoldering subtype with rash and minimal blood involvement, a chronic subtype with higher levels of blood involvement, a lymphoma subtype with lymph node involvement, and an acute subtype with visceral disease, hypercalcemia, and/or elevated lactate dehydrogenase (LDH) [4]. The latter two, more aggressive and lethal subtypes account for the majority of cases. Median survival for ATLL is less than one year, with fewer than four percent of subjects surviving 4 years, despite aggressive chemotherapy [5].

HTLV-1 initially infects T-cells or lymphoid precursors [6] and expresses two viral oncoproteins; Tax, the activator of viral transcription, and helix-basic-zipper protein (HBZ) which is the only viral protein constitutively expressed in all ATLL cells [7]. Tax induces cell proliferation through T-cell receptor (TCR) signaling to nuclear factor κB (NFκB), confers resistance to apoptosis, and promotes genetic instability, all of which contribute to the initiation of T-cell malignancy [8]. These functions are mediated by the effect of Tax on an array of cellular proteins, described as the “Tax interactome” [9]. However, Tax is also highly immunogenic and cells expressing it at high levels are targeted by cytotoxic T lymphocytes (CTL) [10]. This results in selection of ATLL cells that suppress Tax and compensate for loss of Tax by acquiring genetic or epigenetic changes in the Tax interactome [7].

In ATLL cells, somatic mutations are frequently found in genes in the TCR signaling pathway [7]. These mutations precede development of detectable disease, can accumulate in predominant clones over many years, are not found in HTLV-1 infected patients who fail to develop ATLL [11] and signal though NFκB to interferon regulatory factor 4 (IRF4) [12]. The IRF4 gene is the most frequently mutated gene in ATLL, with 1 in 4 patients carrying amplification of the IRF4 gene and 1 in 7 individuals acquiring activating nucleotide variants [7]. Highly expressed in ATLL, IRF4 drives cellular proliferation [13, 14] and is associated with poor prognosis and therapeutic resistance [15, 16]. IRF4 and NFκB have been described as master regulators of transcription in ATLL, forming a coherent feed-forward loop that drives cell proliferation and survival [12]. The downstream targets of IRF4 and its mechanism of action in ATLL pathogenesis remains largely uncharacterized. In one study, knockdown of IRF4 resulted in up-regulation of Th1 transcription factors/cytokines including interferon (IFNγ) and interleukin 7 receptor (IL7R) [17]. We previously reported that the most common IRF4 mutation in ATLL, K59R, led to increased nuclear localization and transcriptional activity of IRF4, with increased expression of several IRF4-target genes, such as *IL2, CCR4*, *MYCN*, and *CTLA4* [18].

Based on these findings, we sought to interrogate IRF4 signaling in vivo and determine if IRF4 represents a therapeutic target for ATLL. IRF4 has been identified as a key driver in other lymphoid malignancies, including multiple myeloma [19]. Lenalidomide, and its predecessor, thalidomide, are proprietary immunomodulatory imide drugs (IMiD) compounds of Celgene Corporation, which have potent anti-inflammatory, anti-angiogenic, and immunomodulatory properties [20]. In addition to multiple myeloma, lenalidomide is approved for use in myelodysplastic syndromes and several non-Hodgkin’s lymphomas [21–23]. Lenalidomide inhibits proliferation of multiple myeloma and primary effusion lymphoma at least partially by repressing IRF4 expression [24–27]. Pomalidomide treatment reduced IRF4 expression in HTLV-1 infected MT2 cells and increased their susceptibility to NK mediated cytotoxicity [28]. However, TLOM1 cells, an ATLL cell line which does not express Tax, were unaffected by pomalidomide. Here we report the sensitivity of ATLL cells to therapies that directly and indirectly target IRF4 and describe downstream effectors of IRF4 expression in CD4 + T-cells in vivo.

## Results

### Proliferation of Tax-negative ATLL cell lines is dependent on IRF4

**Fig. 1:**
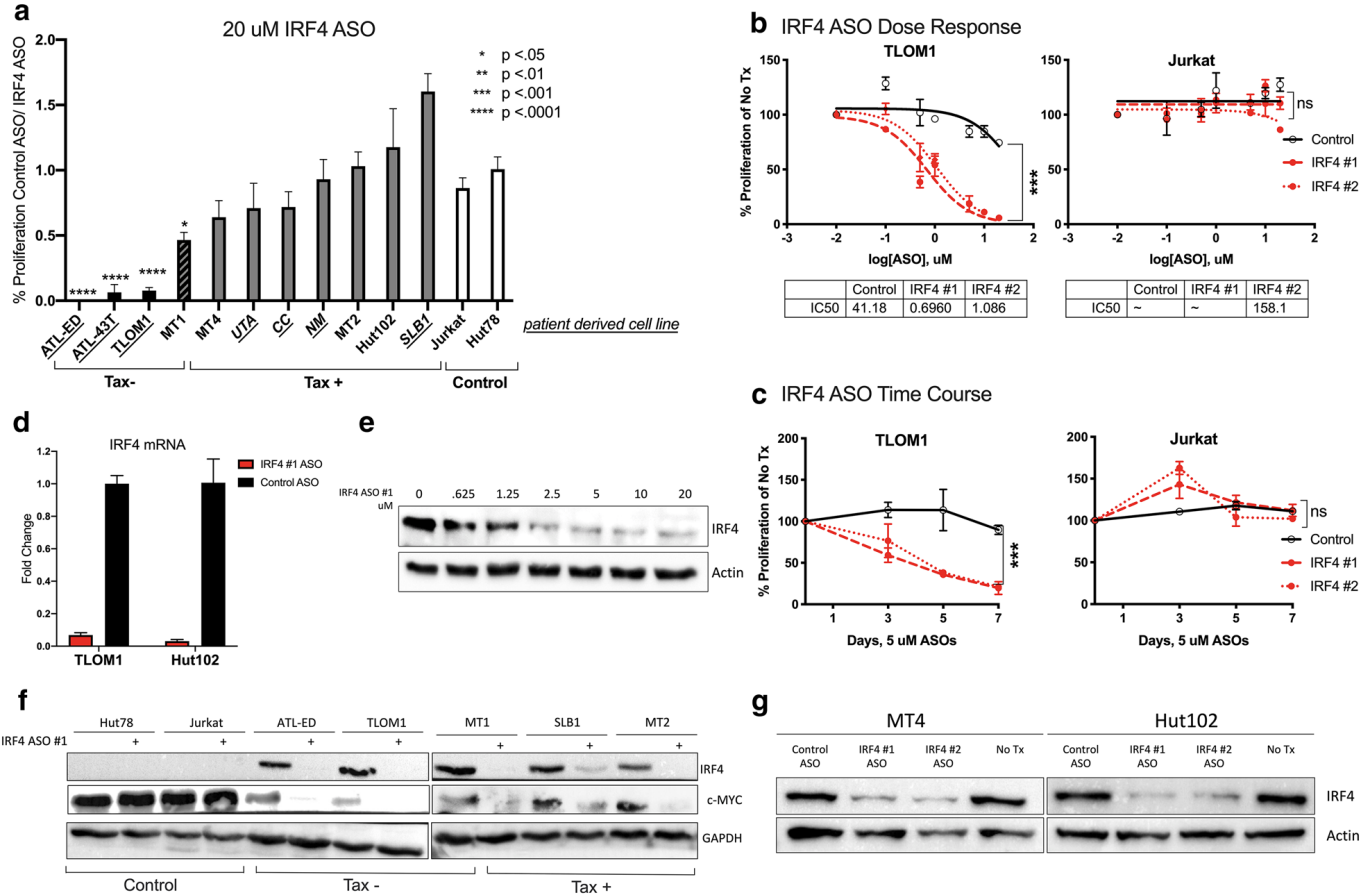
IRF4 antisense oligonucleotides decreased proliferation of adult T-cell leukemia. **a** Resazurin proliferation assay of cells treated for 7 days with 20 μM IRF4#1 normalized to cells treated with Control ASO. **b** Resazurin proliferation assay at 7 days post-treatment. **c** Resazurin proliferation time course of cells treated with 5 μM ASOs. **d** 2 days post-treatment with 10 μM ASOs. **e** ATL-ED cells 4 days post treatment with varying doses of IRF4#1 ASO. Bands were quantified using BioRad software and presented values are normalized to actin. **f** 3 days post-treatment with 20 μM IRF4#1 ASO. **g** 3 days post-treatment with 10 μM ASOs

Antisense oligonucleotides (ASOs) that directly target IRF4 messenger RNA were used to silence IRF4 gene expression in ATLL cell lines, primary patient-derived ATLL cells, and control cell T-cell lines. The IRF4 ASOs are chimeric gapmer nuclease-resistant oligonucleotides with constrained ethyl chemistry that have high affinity, stability, and tolerability; and are currently in multiple myeloma clinical trials [29]. Cell proliferation in the presence of IRF4 ASO or control ASO was measured over the course of 7 days (Fig. 1a). Proliferation of ATLL cells that express the Tax oncogene was largely resistant to the suppression of IRF4, whereas the proliferation of Tax-negative cells was exquisitely sensitive to IRF4 knockdown in a dose-dependent (Fig. 1b) and time-dependent (Fig. 1c) manner. ASO suppressed IRF4 RNA (Fig. 1d) and protein (Fig. 1e) equally well in Tax-negative and Tax-expressing cells (Fig. 1f) and two different IRF4 ASOs showed similar results (Fig. 1g). Taken together, these data suggest that IRF4 is essential for proliferation of ATLL cells that do not express the viral oncogene Tax.

### Proliferation of ATLL cells is suppressed by lenalidomide

**Fig. 2:**
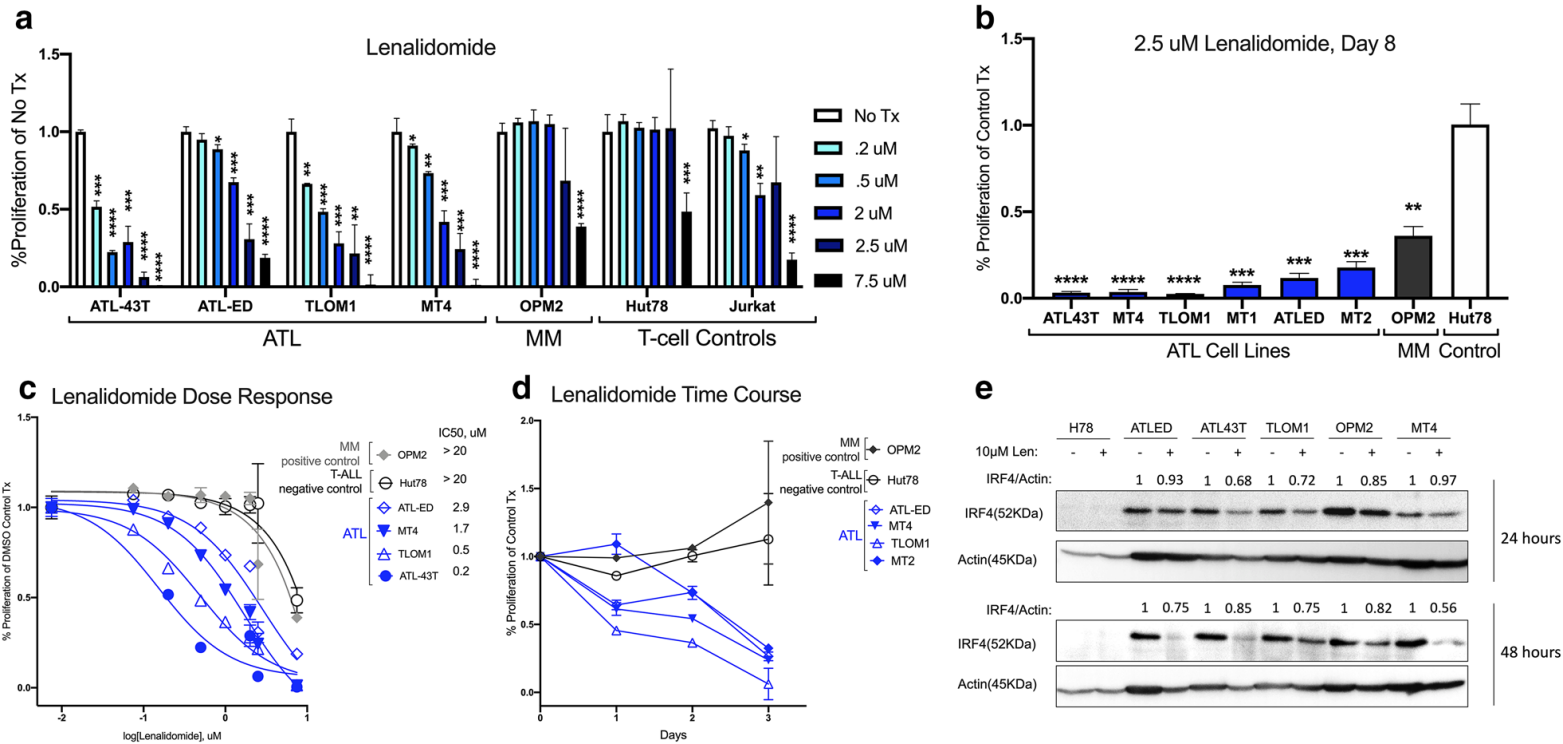
Lenalidomide decreases proliferation and reduces IRF4 expression in ATLL. **a** Resazurin proliferation assay of cells treated for 2 days with varying doses of lenalidomide normalized to cells treated with DMSO control. Two-tailed T-tests of each dose compared to DMSO were conducted for each cell line. **b** Resazurin proliferation assay at 8 days post-treatment with 2.5 μM lenalidomide. Two-tailed T-tests were conducted for each cell line compared to Hut78 control T-cells. **c** Dose response curves at 4 days post-treatment. IC50s were calculated with Prism Graphpad software. **d** Proliferation time course of cells treated with 1 μM lenalidomide. **e** Western blot of cells treated for 24 h with 10 and 20 μM lenalidomide. Bands were quantified using BioRad software and presented values are normalized to actin

By targeting cereblon, lenalidomide affects the expression of a variety of transcription factors including IRF4 and downstream targets like Myc [27, 30]. Treatment of ATLL cells with lenalidomide resulted in striking suppression of proliferation in a dose dependent manner (Fig. 2a). Within two days of exposure to lenalidomide, ATLL cells were more sensitive than other T-cell lines, as well as OPM2 cells, a multiple myeloma cell line. The proliferation of all ATLL cells tested was suppressed over the course of 8 days (Fig. 2b). Unlike the IRF4 ASO, the effect of lenalidomide was independent of Tax expression. MT2 and MT4 cells, which express Tax, were suppressed along with the Tax-negative ATLL cells (Fig. 2a–d). Like IRF4 ASO, lenalidomide treatment resulted in decreased IRF4 protein (Fig. 2e). In some cell lines, like MT2, MT4, and ATL43T, the effect of lenalidomide on proliferation was more potent than that of IRF4 ASO. In other cell lines, like TLOM1 and OPM2, the opposite was true (Additional file 1: Figure S1).

**Fig. 3:**
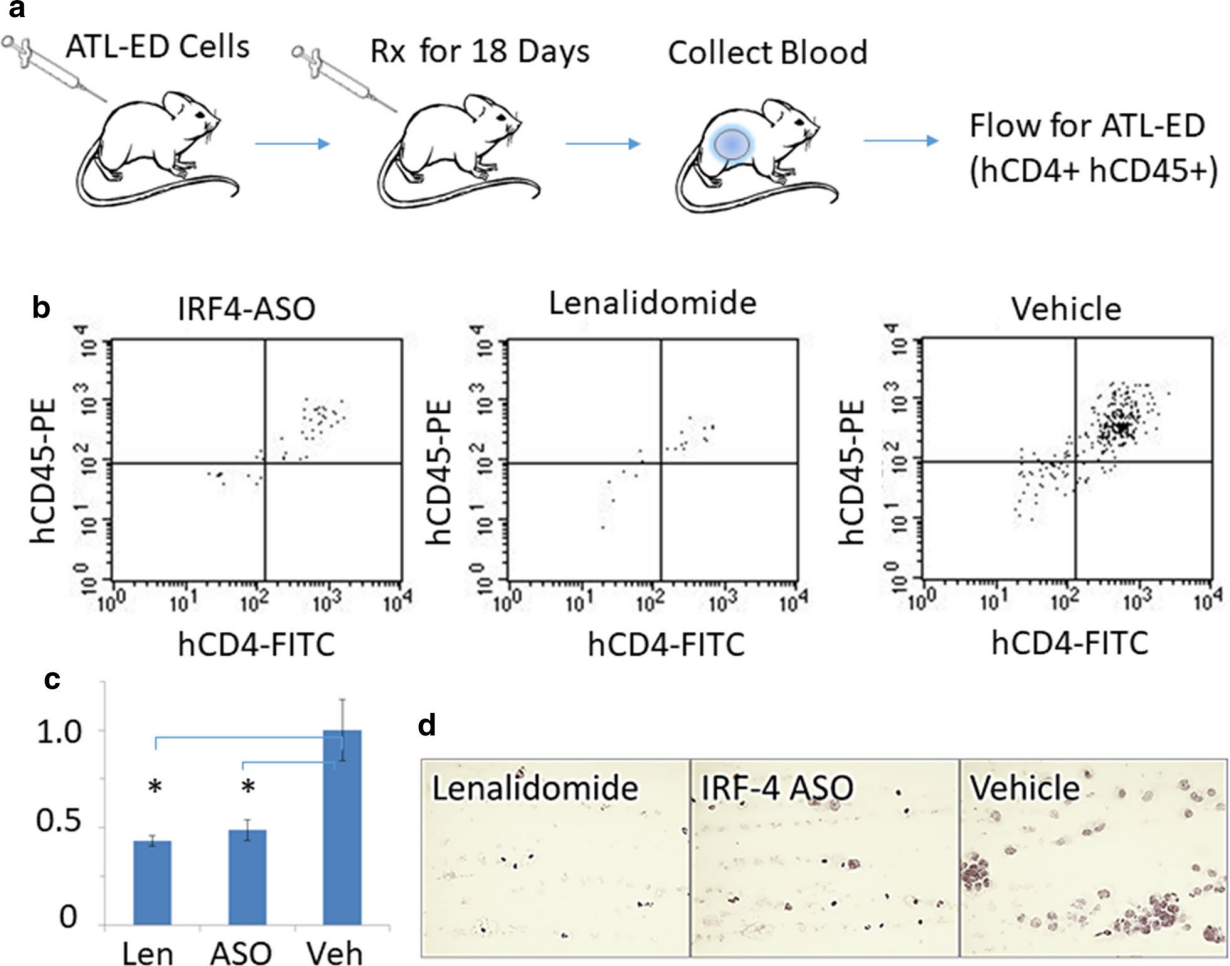
IRF4 ASO and lenalidomide decrease ATLL tumor burden in vivo. On Day 0, NSG mice received an intraperitoneal injection of 10 million ATL-ED cells. Mice were treated with 12 doses of IRF4 ASO (n = 2), lenalidomide (n = 2), or PBS (n = 3) over 18 days. Mice were euthanized on day 18 and peripheral blood was collected. **a** Schematic of experimental design. **b**, **c** Representative flow cytometry data of human ATLL cells in the peripheral blood (hCD45 + hCD4 +) and quantitation of the flow data set. **d** Representative blood smears of each treatment group

To evaluate the efficacy of ASO and lenalidomide in vivo, ATL-ED cells were injected intraperitoneally (ip) into NSG-KIT mice (Fig. 3a). Over the course of 18 days, mice received ip. injections of ASO, lenalidomide, or vehicle (sterile saline). Fewer ATL-ED cells were present in the peripheral blood at the time of sacrifice in the treated animals compared to vehicle control (Fig. 3b–d). These data suggest that lenalidomide is capable of significantly suppressing the proliferation of ATLL cells both in vitro and in vivo.

### IRF4 signaling in T-cells

**Fig. 4:**
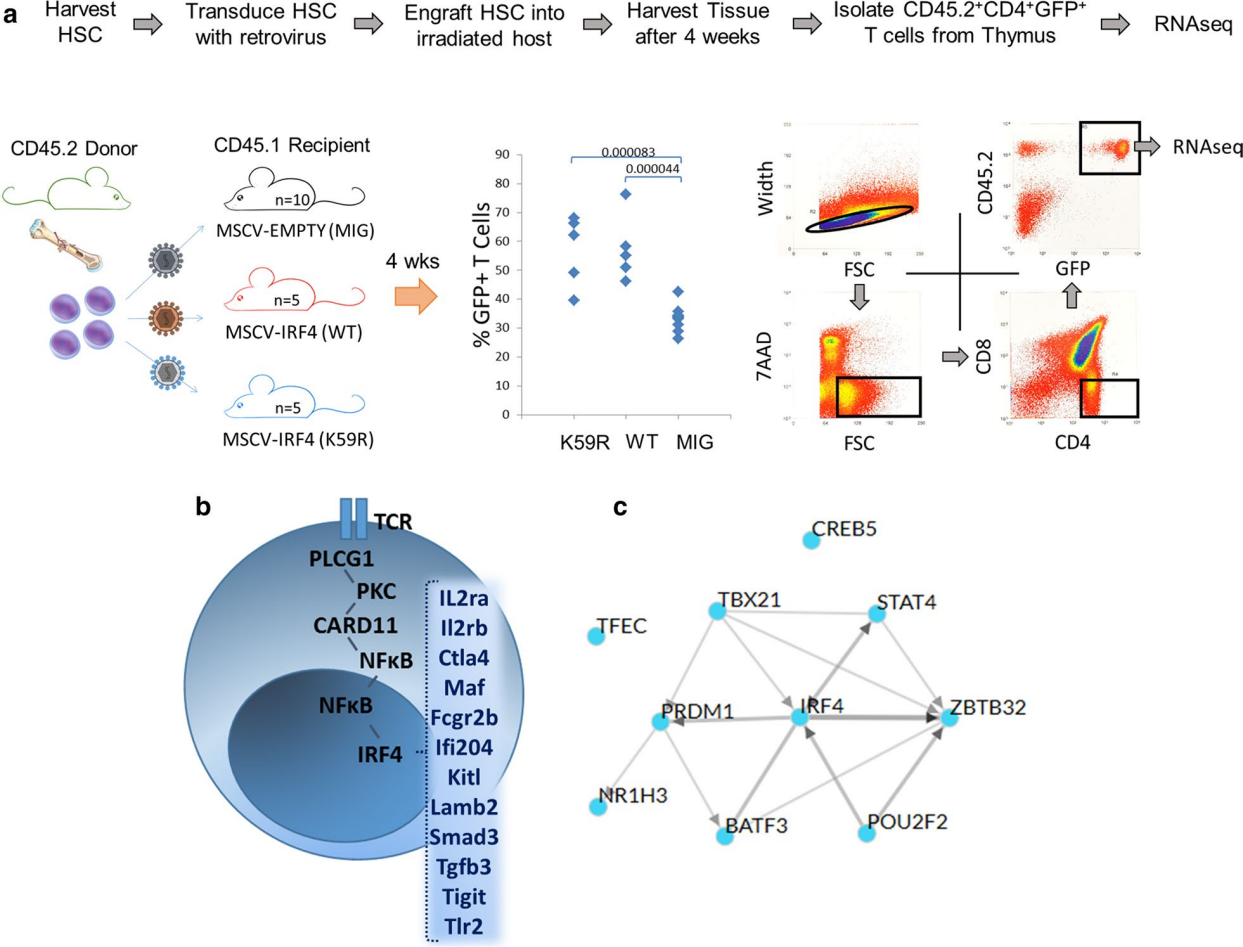
Identification of IRF4 regulated genes in CD4 + T-cells in vivo. **a** CD45.1 HSC were transduced with IRF4 expressing retroviral vectors and engrafted into CD45.2 hosts. Peripheral blood was collected and mice were euthanized after 4 weeks. RNAseq was performed on CD45.1, GFP + , CD4 + T-cells purified by FACS from pooled thymocytes. **b** Genes elevated in CD4 + T-cells expressing IRF4 compared to CD4 + T-cells with empty vector. **c** Transcription factor networks associated with gene list identified using the ChEA3 Transcription Factor Local Network Analysis of Top Gene Hits (https://amp.pharm.mssm.edu/chea3/#top)

To understand the mechanisms governing the role of IRF4 in the pathogenesis of ATLL and to discover potential biomarkers for ATLL therapy, we sought to identify downstream targets of IRF4 signaling in CD4 + T-cells in vivo. Using GFP-tagged retroviral vectors, murine hematopoietic stem cells (HSC) from CD45.2 mice were transduced with IRF4 (WT), IRF4 (K59R), or empty vector and engrafted into CD45.1 hosts. Overexpression of IRF4 (WT) or IRF4 (K59R) in the bone marrow of host mice led to marked expansion of T lymphoid cells, but no alteration of B lymphoid or myeloid cell populations (Fig. 4a) [18]. After 4 weeks, RNAseq was performed on GFP + CD45.2 + CD4 + CD8 − T-cells harvested from the thymus (3 mice for each condition). One hundred and five IRF4 downstream targets were up-regulated in T-cells from both IRF4 (WT) and IRF4 (K59R) mice compared to T-cells harvested from control mice (Additional file 1: Figure S2). The genes associated with IRF4 (WT) and IRF4 (K59R) expression included *CASP1, CHST2, CLEC12A, CTLA4, FCGR2B, IFI204, IL2RA, IL2RB, KITLG, LAMB2, LY86, MAF, SMAD3, TGFB3, THSD1, TIGIT*, *TOX2,* and *TLR2*. Many of these genes, such as CTLA4 and TOX2, are also up-regulated in ATLL samples (Additional file 1: Figure S3). In addition to IRF4, transcription factors most closely associated with the genes identified in this study included BATF3 and PRDM1, known IRF4 binding partners (Fig. 4c). These data reveal that overexpression of IRF4 in HSC results in expansion of the T cell compartment and the expression of factors characteristic of ATLL and capable of promoting lymphoid proliferation downstream of TCR signaling.

## Discussion

Therapeutic nucleic acid-based approaches, including ASOs, offer the potential to yield drugs, based on gene sequence information alone, for targets that have proven to be intractable to alternative drug modalities, such as transcription factors like IRF4 [31]. Therapeutic ASOs have common chemical and biological properties and are generally safe and well-tolerated in the clinic. This has led to several recent new drug approvals including Tegsedi™, Waylivra™ and the blockbuster drug Sprinraza™ for the treatment of patients with the devastating neurodegenerative disease spinal muscular atrophy [32]. Continued efforts to improve upon the stability and potency of ASOs has resulted in the discovery of a next-generation class of ASOs that employ 2′-4′ constrained ethyl (cEt) residues and exhibit significantly enhanced in vitro and in vivo potency compared to earlier generation ASO molecules [33]. More recently, these next-generation cEt-containing ASOs targeted to previously undruggable tumor cell targets have shown therapeutic promise for the treatment of cancer. The first cEt-containing ASOs to be evaluated clinically are AZD9150/Danvatirsen, which targets the transcription factor STAT3, and ION-560131/AR_RX,_ that targets all forms of the androgen receptor [33, 34]. Both of these novel therapeutics have demonstrated good tolerability and efficacy in clinical trials. Danvatirsen has shown strong single-agent antitumor activity in several cancer patient populations in early clinical studies. Moreover, ION-560131/AR_RX_ has shown encouraging monotherapy clinical efficacy in late-stage metastatic prostate cancer patients.

There are no effective therapies for ATLL, the median survival time for this malignancy is measured in months, and in the U.S., minorities are disproportionately affected. Therapeutic options that effectively target ATLL cells are urgently needed. Recent breakthroughs in our understanding of the genomics of ATLL have revealed that this malignancy selects for constitutively active TCR signaling. The TCR signaling pathway is initially activated by the Tax oncogene in early stages of HTLV-1 infection, which drives transformation and immortalization. In HTLV-1 transformed T-cells, the TCR provides a signal driving transcription through master regulators IRF4 and NFκB in a feed-forward loop. Later, somatic activating mutations in the TCR pathway genes themselves replace the need for Tax, promoting immune evasion and disease progression. Lenalidomide, an FDA approved drug currently used to treat multiple myeloma and other lymphoid malignancies, indirectly suppresses IRF4. As a pre-requisite for a clinical trial, these studies were performed to determine if lenalidomide treatment affects the proliferation of ATLL cells in tissue culture and animal models, and to identify the downstream targets of IRF4 in CD4 + T-cells in vivo.

First, the ATLL cells tested were sensitive to lenalidomide. Both dose-dependent and time- dependent inhibition of ATLL cells was more pronounced than that of other sensitive control cell lines, including one derived from multiple myeloma. Lenalidomide was efficacious against ATLL both in cell culture and in mouse xenograft experiments. The mechanism underlying the effect of lenalidomide on ATLL cells is not clear. While lenalidomide suppresses IRF4 expression and ATLL cell proliferation, targeted knockdown of IRF4 using ASO only suppressed proliferation of the Tax-negative subset of ATLL cells. This suggests that IRF4 is essential for the proliferation of Tax-negative ATLL cells and also suggests that lenalidomide suppression of ATLL cells is mediated by more than one mechanism, as has been described in multiple myeloma.

Second, the IRF4 ASO results indicate that ATLL cell lines that retain expression of the viral oncogene Tax are able to proliferate in the absence of IRF4, whereas IRF4 is essential for proliferation of ATLL cells that no longer express Tax. It is unclear if IRF4 dependency in Tax-negative ATLL cells is associated with acquisition of specific mutations or whether it can be bypassed by overexpression of the Tax oncogene. This discovery exposes a previously unknown relationship between Tax and IRF4. Since Tax expression is a strong selective disadvantage during lymphomagenesis and is typically silenced, ATLL cells in the context of tumor immunity may be more sensitive to IRF4-targeted therapies than the same cells cultured ex vivo or as xenografts in immunodeficient mouse models. A pilot study of lenalidomide in a small cohort of Japanese patients with ATLL showed significant clinical response [35].

Third, while constitutive TCR signaling through NFκB to IRF4 regulates ATLL, the downstream targets of IRF4 that drive ATLL cell proliferation are not known. Identifying these downstream effectors will not only provide mechanistic insights into ATLL pathogenesis but will also provide biomarkers for disease progression and additional therapeutic targets. Overexpression of IRF4 in HSC in the bone marrow of mice promoted the expansion of T-cells in the peripheral blood within 4 weeks. Examination of the transcriptome of these expanded CD4 + T-cells in the thymus revealed a subset of genes likely to be involved in T-cell proliferation and ATLL progression. Half of the genes identified were previously identified by Krishnamoorthy et al. as factors regulated by IRF4 downstream of TCR signaling in T-cells in a transgenic model [36]. Notably, several of the genes encode ATLL cell surface markers (IL2RA also known as CD25), checkpoint regulators (CTLA4, TIGIT), and key regulators of T-cell proliferation (MAF, KITLG, TGFB3). Several other of the IRF4 regulated factors we identified have been implicated in ATLL pathogenesis. TNSF11 encodes RANKL which mediates hypercalcemia characteristic of ATLL [37]. JAG1 is a Notch ligand, and Notch signaling is activated in ATLL [38]. Our previous studies showed that ETS1 is a target of Tax transactivation [39] and that BLK is overexpressed in ATLL [40]. A previous study examined IRF4-induced gene expression in TCR-driven CD8 + T-cells from wild type and knockout animals, and identified key regulators: PRDM1 (which encodes Blimp-1), eomesodermin (EOMES), runt-related transcription factor 3 (Runx3), TCF7, and IL7R [41]. These genes may represent new therapeutic targets for ATLL and may be useful as biomarkers in a clinical trial of Lenalidomide.

## Conclusions

Our recent experience testing the efficacy of checkpoint inhibitors in ATLL highlights the powerful role of immunity in ATLL pathogenesis and progression, and the desperate need for targeted therapies [6]. Elucidating the mechanism by which TCR signaling through IRF4 is driving ATLL is an essential step in identifying and targeting these critical factors. The IRF4 ASO has advanced into a clinical trial in multiple myeloma (NCT04398485). Lenalidomide has advanced in a clinical trial with combination chemotherapy in ATLL (NCT04301076).

## Methods

### Cell culture

TLOM1, MT1, and ED40515 Tax-negative ATL cell lines were obtained from Edward Harhaj (Penn State University) and OPM2 cells were kindly provided by Kareen Azab (Washington University). The Hut102, ATL-ED, ATL-43T, and SLB1 cell lines were gifts from Patrick Green (Ohio State University). Jurkat, 293T, MT2, and MT4 cells were obtained from the National Institutes of Health AIDS Repository. Cell lines were maintained at 37 °C and 5% CO_2_ in complete medium supplemented with 10% fetal bovine serum, 4 mM L-glutamine, 100 units/ml penicillin, and 100 μg/ml streptomycin. PBMCs and T-cell lines, including Jurkat, MT2, MT4, TLOM1, MT1, and ED40515 cells, were maintained in complete RPMI medium.

### Therapeutics

ASOs were from Ionis Pharmaceutical. Lenalidomide was purchased from AdooQ Bioscience (A10522-10 mM-D) presolubilized in DMSO. Lenalidomide was stored at room temperature, never frozen, and discarded after 2 weeks.

### Western Blotting

Cells were lysed in Tris–HCl, pH 6.8, with 2% Igepal CA630, 2% SDS, 10 mM sodium glycerophosphate, 10 mM sodium fluoride, 2.5 mM sodium pyrophosphate, 1 mM sodium orthovanadate, and EDTA-free protease inhibitor mixture (Roche Applied Science/Sigma). Lysates were sonicated on ice for 3 cycles of 30 s on 30 s off. Protein concentration was determined using the bicinchoninic acid assay. Equal amounts of protein (40 ug) were mixed with 4 × Sample buffer (25% Tris–HCL pH 6.8, 13% water, 0.8% SDS, 40% Glycerol, bromophenol blue, 20% 2-Mercapto ethanol) and briefly boiled before loading on to 10% SDS-PAGE gel. Buffers for gel, transfer, and washing steps followed protocols described by Proteintech. Blots were blocked for 1 h in 5% protease-free BSA at room temperature. Antibodies were diluted in 5% protease-free BSA. Blots were imaged by enhanced chemiluminescence using a Chemidoc imager (Bio-Rad) and HRP substrate Clarity™ Western ECL Substrate (1705060) (Bio-rad). The following antibodies were used for Western blots: mouse primary monoclonal antibody to IRF4 (sc-48338) and to c-MYC (sc-40), rabbit primary monoclonal antibody to β-Actin (cell signaling 13E5), and anti-rabbit monoclonal antibody (cell signaling 4970).

### Proliferation assay

Cells were plated at 10,000 cells per well in 200 μL in a round bottom 96 well plate. Plates were covered and incubated at 37 °C. At the specified time points, 20 μL of AlamarBlue (ThermoFisher Scientific) was added to the wells to achieve a final concentration of 0.15 mg/ml. Plates were placed back in the incubator and absorbance was read after 2 h on the SpectraFluor Plus Spectrophotometer (Tecan).

### Quantitative PCR

RNA was extracted using TRIzol reagent. Total RNA was used as template for reverse transcription and amplification of cDNAs in a three-step amplification protocol using the iTaq Universal SYBR Green one-step kit (Bio-Rad) on a Bio-Rad CFX Connect real-time system with an extension temperature of 65 °C. Exon-exon junction–spanning primers were designed to reduce background from genomic DNA. The following primers were used for qRT-PCR: HGPRT1 (forward), GCCCTGGCGTCGTGATTAGT; HGPRT1 (reverse), GTTGACTGGTCATTACAATA; IRF4 (forward), TTGGCGTTCTCAGACTGCCG; IRF4 (reverse), AACGCTTGCAGCTCTGACAA. Cycle number (Ct value) for each measured transcript was converted to ratios of transcript abundance by taking the antilog to the base 2 of (-1 × Ct) value for each transcript and dividing that by the antilog to the base 2 of (−1 × Ct) value for hypoxanthine guanine phosphoribosyl transferase based on the assumption of a 100% amplification efficiency.

### In vivo studies

All animal studies were performed in accordance with human research protocols approved by the division of comparative medicine and the Institutional Animal Care and Use Committee (IACUC; Animal Welfare Assurance #A-3381-01) at Washington University in Saint Louis. 4 month old NSG mice were injected intraperitoneally with 10^6^ ATL-ED in 150 μL of PBS. Mice were treated with intraperitoneal injections with two days on one day off for 12 doses over 18 days. IRF4 ASO treatment included 50 mg/kg suspended in 200 μL saline based on recommendations from the manufacturer (Ionis pharmaceuticals). Lenalidomide treatment included 50 mg/kg suspended in 200 μL saline. Control mice were treated with 200 μL PBS. ASOs and lenalidomide were initially solubilized in DMSO and diluted to a final dosed concentration of less than 10% DMSO. Mice were sacrificed at day 18 post tumor injection and blood smears and flow cytometry were used to assess peripheral blood leukemia burden.

### Transduction of murine hematopoietic stem cells and transplantation into recipient mice

150 mg/kg 5-fluorouracil was administered to Donor C57/B6 CD45.1 mice 6 days before bone marrow harvest. Harvested marrow was enriched for Sca-1^+^ cells by autoMACS (Miltenyi). Sca-1 + enriched cells were adjusted to 5 × 10^5^ cells/ml in stem cell media cocktail containing Stempro34 serum-free medium supplement (Gibco), 10 ng/ml stem cell factor (R&D Systems), 100 ng/ml mouse thrombopoietin, 100 units/ml penicillin, 100 μg/ml streptomycin, 4 μg/ml Polybrene, and 2 mm glutamine. MSCV particles were generated in 293T cells by calcium phosphate–mediated co-transfection of pCL-ECO packaging plasmid with either the empty MSCV-IRES-GFP retroviral vector or vector with coding sequence for WT IRF4 or K59R mutant IRF4 inserted by LR clonase recombinational cloning and harvested from the medium by ultracentrifugation. Sca-1^+^ enriched donor marrow cells were transduced with MSCV retroviral particles by spin-inoculation at 250 × *g* at room temperature for 2 h, followed by incubation for 1 h at 37 °C, and then transplanted by retro-orbital injection into lethally irradiated (9.5 grays) C57/B6 CD45.2 recipients (*n* = 10 for empty vector and 5 each for WT IRF4 and K59R mutant IRF4). Four weeks after engraftment, peripheral blood was collected, and FACS was used to determine the abundance of CD45.1^+^, GFP^+^ myeloid cells (Gr-1 + , Mac-1 +), B cells (B220^+^), and T-cells (CD4^+^, CD8^+^). Mouse strains used for this study were obtained from the Jackson Laboratory (Bar Harbor, ME).

### RNA sequencing

Total RNA was obtained from flow sorted GFP + CD45.2 + CD4 + CD8- T-cells harvested from the thymus of three mice in each group (empty vector / MIG; wild-type IRF4/ WT; and mutant IRF4 / K59R) using RNeasy (QIAGEN). RNA was submitted to the Genome Technology Access Center at Washington University for RNA sequencing (RNAseq) analysis, processed for low quantity input with Takara-Clontech SMARTer, and sequenced on a NovaSeq S4, 2 × 150 platform. Reads were aligned to the reference genome using STAR, and gene counts were derived from uniquely aligned reads using Subread:featureCount.BAM files for RNAseq and probe capture DNA were submitted to the Sequence Read Archive Database under BioProject accession number PRJNA658553.

### Statistics

Data were analyzed by two-tailed unpaired Student’s t test for samples of equal variance for statistical significance. Error bars specify standard error. Statistics were conducted using Prism GraphPad 8.0.

## Supplementary information

**Additional file 1: Figure S1.** Sensitivity of cell lines to combination of lenalidomide and IRF4 ASO. Cells were treated for 2 days with either 10 uM IRF4 ASO, 2.5 uM lenalidomide, Combination, or Control ASO + DMSO Treatment. Resazurin proliferation assay were conducted and values are presented as normalized to control treatment for each cell line.** Figure S2.** IRF4 gene targets up-regulated in primary T-cells. Total RNA was obtained from flow sorted GFP+ CD45.2+ CD4+ CD8- T-cells harvested from the thymus of three mice in each group and submitted to the Genome Technology Access Center at Washington University for RNA sequencing (RNAseq) analysis. Normalized gene counts for each of the 9 samples were divided by the average number of normalized counts for the 3 MIG samples. Colors indicate fold increase for each gene in each sample over average control (MIG) value for each gene. Genes listed are those that were expressed (>10 reads) and elevated (>2 fold) in cells expressing WT IRF4 and IRF4 K59R.** Figure S3.** IRF4 gene targets confirmed in primary ATL. Data from an ATLL gene expression microarray study was downloaded from the Gene Expression Omnibus at the NCBI (GEO accession: GSE33615). In the original study, RNA was extracted from PBMCs isolated from patients with acute (n=26), chronic (n=20), lymphomatous (n=1), and smoldering (n=4) ATLL, and compared to RNA obtained from CD4+ cells from 21 normal subjects. In this study, values for CTLA4 and TOX2 were normalized to actin (ACTB) then represented as fold-Patient 10 (a smoldering ATLL sample with the lowest proviral load in the study). A) Correlation of TOX2 and CTLA4 to IRF4 expression in ATLL. B) Expression of TOX2, CTLA4, and IRF4 in IRF4 HI ATLL samples compared to normal T-cells. C) Expression of TOX2, CTLA4, and IRF4 in acute, chronic, lymphoma, and smoldering subtypes of ATLL.

## Data Availability

Transcriptomic data were submitted to Sequence Read Archive Database. All other data sets are available upon request from the corresponding author.
