# Supplementary material for: Interferon regulatory factor 4 as a therapeutic target in adult T-cell leukemia lymphoma
Source: Retrovirology. 2020 Aug 28;17:27. doi: 10.1186/s12977-020-00535-z (PMC7456374; doi:10.1186/s12977-020-00535-z)
Supplement: Supplementary file 1 — Additional file 1: Figure S1. Sensitivity of cell lines to combination of lenalidomide and IRF4 ASO. Cells were treated for 2 days with either 10 uM IRF4 ASO, 2.5 uM lenalidomide, Combination, or Control ASO + DMSO Treatment. Resazurin proliferation assay were conducted and values are presented as normalized to control treatment for each cell line. Figure S2. IRF4 gene targets up-regulated in primary T-cells. Total RNA was obtained from flow sorted GFP+ CD45.2+ CD4+ CD8- T-cells harvested from the thymus of three mice in each group and submitted to the Genome Technology Access Center at Washington University for RNA sequencing (RNAseq) analysis. Normalized gene counts for each of the 9 samples were divided by the average number of normalized counts for the 3 MIG samples. Colors indicate fold increase for each gene in each sample over average control (MIG) value for each gene. Genes listed are those that were expressed (>10 reads) and elevated (>2 fold) in cells expressing WT IRF4 and IRF4 K59R. Figure S3. IRF4 gene targets confirmed in primary ATL. Data from an ATLL gene expression microarray study was downloaded from the Gene Expression Omnibus at the NCBI (GEO accession: GSE33615). In the original study, RNA was extracted from PBMCs isolated from patients with acute (n=26), chronic (n=20), lymphomatous (n=1), and smoldering (n=4) ATLL, and compared to RNA obtained from CD4+ cells from 21 normal subjects. In this study, values for CTLA4 and TOX2 were normalized to actin (ACTB) then represented as fold-Patient 10 (a smoldering ATLL sample with the lowest proviral load in the study). A) Correlation of TOX2 and CTLA4 to IRF4 expression in ATLL. B) Expression of TOX2, CTLA4, and IRF4 in IRF4 HI ATLL samples compared to normal T-cells. C) Expression of TOX2, CTLA4, and IRF4 in acute, chronic, lymphoma, and smoldering subtypes of ATLL. [file 12977_2020_535_MOESM1_ESM.pdf]

FIG S1

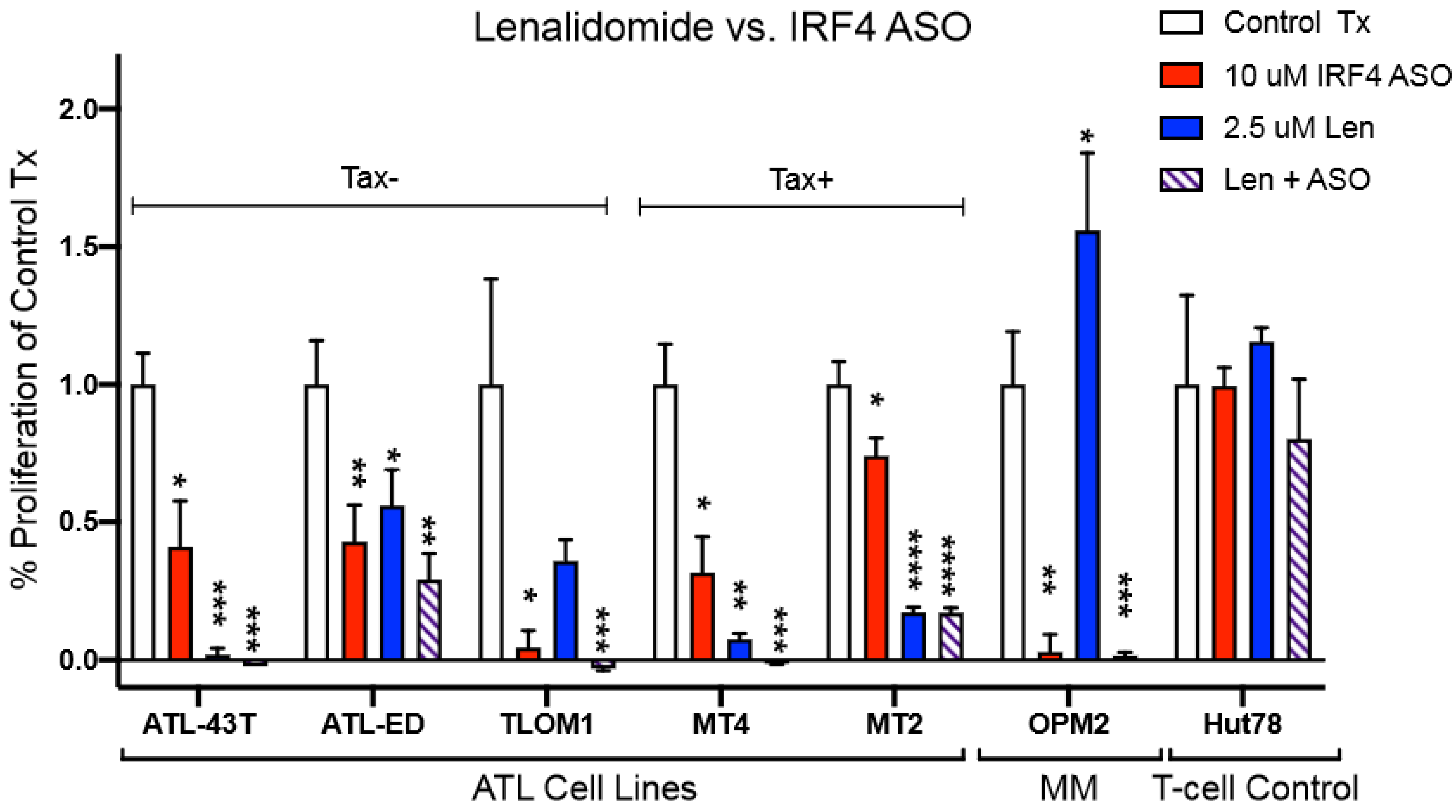

Sensitivity of cell lines to combination of Lenalidomide and IRF4 ASO. Cells were treated for 2 days with either 10 uM IRF4 ASO, 2.5 uM lenalidomide, Combination, or Control ASO + DMSO Treatment. Resazurin proliferation assay were conducted and values are presented as normalized to control treatment for each cell line.

FIG S2

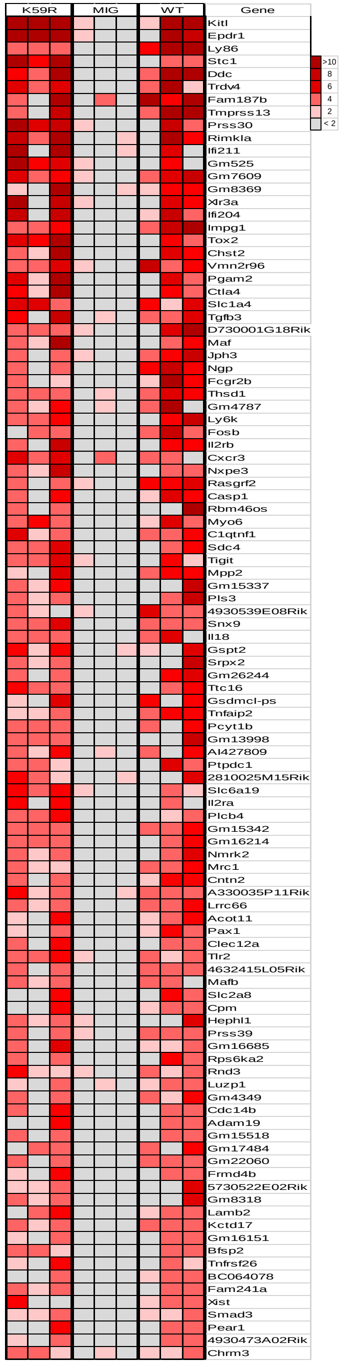

IRF4 gene targets up-regulated in primary T cells

Total RNA was obtained from flow sorted GFP+ CD45.2+ CD4+ CD8- T cells harvested from the thymus of three mice in each group and submitted to the Genome Technology Access Center at Washington University for RNA sequencing (RNAseq) analysis. Normalized gene counts for each of the 9 samples were divided by the average number of normalized counts for the 3 MIG samples. Colors indicate fold increase for each gene in each sample over average control (MIG) value for each gene. Genes listed are those that were expressed (>10 reads) and elevated (>2 fold) in cells expressing WT IRF4 and IRF4 K59R.

FIG S3

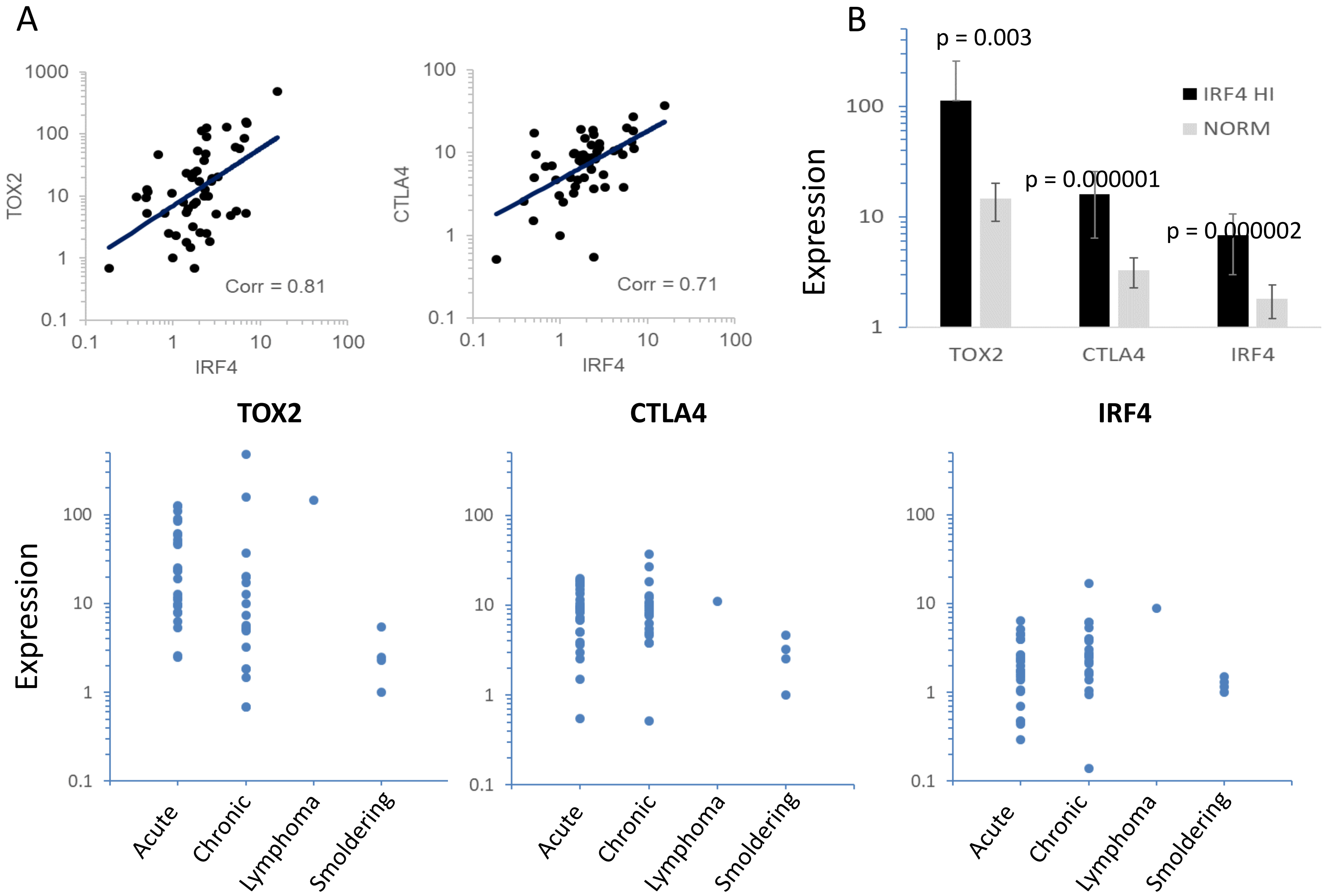

IRF4 gene targets confirmed in primary ATL. Data from an ATLL gene expression microarray study was downloaded from the Gene Expression Omnibus at the NCBI (GEO accession: GSE33615). In the original study, RNA was extracted from PBMCs isolated from patients with acute (n=26), chronic (n=20), lymphomatous (n=1), and smoldering (n=4) ATLL, and compared to RNA obtained from CD4+ cells from 21 normal subjects. In this study, values for CTLA4 and TOX2 were normalized to actin (ACTB) then represented as fold-Patient 10 (a smoldering ATLL sample with the lowest proviral load in the study). A) Correlation of TOX2 and CTLA4 to IRF4 expression in ATLL. B) Expression of TOX2, CTLA4, and IRF4 in IRF4 HI ATLL samples compared to normal T cells. C) Expression of TOX2, CTLA4, and IRF4 in acute, chronic, lymphoma, and smoldering subtypes of ATLL.
